# Classification of Vascular Hotspots and Micro-Vessel Flow Velocity Waveforms in Low-Grade Squamous Intraepithelial Lesions and HPV Condyloma of the Cervix

**DOI:** 10.3390/diagnostics12102390

**Published:** 2022-10-01

**Authors:** Yi-Cheng Wu, Ching-Hsuan Chen, Yi-Li Ko, Chiou-Chung Yuan, Peng-Hui Wang, Woei-Chyn Chu

**Affiliations:** 1 Department of Biomedical Engineering, National Yang-Ming Chiao-Tung University, Taipei 112304, Taiwan; 2 Department of Obstetrics and Gynecology, Ton Yen General Hospital, Hsinchu 302048, Taiwan; 3 Taiwan IVF Group Center for Reproductive Medicine & Infertility, Hsinchu 302053, Taiwan; 4 Department of Obstetrics and Gynecology, Fuyou Branch, Taipei City Hospital, Taipei 100027, Taiwan; 5 Nursing Department, Fu Jen Catholic University, New Taipei 242062, Taiwan; 6 Department of Obstetrics and Gynecology, Cheng Hsin General Hospital, Taipei 112201, Taiwan; 7 Department of Obstetrics and Gynecology, Taipei Veterans General Hospital, Taipei 112201, Taiwan; 8 Institute of Clinical Medicine, National Yang-Ming Chiao-Tung University, Taipei 112304, Taiwan; 9 Female Cancer Foundation, Taipei 104509, Taiwan; 10 Department of Medical Research, China Medical University Hospital, Taichung 404327, Taiwan

**Keywords:** power Doppler ultrasound, human papillomavirus, condyloma, low-grade squamous intraepithelial lesions

## Abstract

To assess hotspot micro-vessel flow velocity waveforms in human papillomavirus (HPV) cervical infections using transvaginal power Doppler ultrasound (TV-PDU) and to explore the associations of these sonographic parameters with HPV condyloma and low-grade squamous intraepithelial lesions (LSIL) of the cervix. A total of 39 patients with cervical HPV infections with abnormal cytology and colposcopy results (26 cases of LSIL; 13 cases of HPV condyloma) were enrolled to assess the vascular classification of the cervix and micro-vessel flow velocity using TV-PDU before treatment; 40 individuals with a pathologically normal cervix were used as the control group; seven parameters were measured, including vascular grading classification (Class I, Class II, and Class III), lowest pulsatility index (PI), resistance index (RI), peak systolic velocity (PS), end-diastolic velocity (ED), time average maximum velocity (TAMV), and the vascular index (VI = PS/ED). According to vascular classification, most LSILs were class I (69.2%, 18/26), followed by class II (26.9%, 7/26) and class III (3.8%, 1/26). Most HPV condylomas were class I (92.3%, 12/13), and one was class II (7.7%, 1/13). PI, RI, VI (*p* < 0.0001), and the PSs (*p* < 0.05) were significantly lower in these cases than in the controls. The ED and TAMV were not significantly different between the patients and controls (*p* = 0.4985 and *p* = 0.1564). No sonographic parameter was significantly different between LSIL and HPV condyloma. The mean PI, RI, and VI were significantly lower in LSIL than in the controls. For HPV condyloma, a PI of 1.07 had an 84.6% sensitivity, 85.0% specificity, and an AUC of 88.8%; for LSIL, a PI of 1.08 had a 100% sensitivity, 85% specificity, and an AUC of 94.2%; for HPV infection (HPV condyloma + LSIL), a PI of 1.08 had a 94.9% sensitivity, 85% specificity, and an AUC of 92.4%. Hotspot vascular classification and micro-vessel flow velocity waveforms may provide a potential practical method for the auxiliary diagnosis of cervical HPV infection. The PI may represent a valuable index for distinguishing the micro-vessel flow velocity waveforms in LSIL and HPV condyloma. Since the case numbers were limited in the current study, further validation is needed.

## 1. Introduction

Human papillomavirus (HPV) is a DNA virus from the Papillomaviridae family, containing more the 450 genomes of almost distinct types which have been isolated and sequenced [1,2]. Many HPV infections cause no apparent symptoms and can be considered part of the normal microbial epithelial flora [1]. It is estimated that 90% of infections spontaneously regress or clean out within two years [3,4,5]. Squamous intraepithelial lesions (SIL) of the cervix—abnormal growths of epithelial cells on the cervix surface—are usually caused by HPV infection [1]. Still, symptoms or abnormal cytology changes might not develop until a long time after infection. Colposcopy with/without endocervical sampling remains a gold standard for the diagnosis of these precancerous lesions of the cervix [6]. According to the Bethesda system, SIL can be graded as LSIL (low-grade squamous intraepithelial lesion) or HSIL (high-grade squamous intraepithelial lesion) [7]. LSIL is also called mild dysplasia of the cervix, and HSIL is regarded as moderate or severe dysplasia. These abnormal areas of tissue may become cancerous. Numerous studies have demonstrated that angiogenesis and the vascularity of cervical cancer correlate with the individual tumor characteristics and prognostic factors for recurrence [8,9,10,11,12,13,14,15]. 

The main objective of this study was to attempt to perform a detailed assessment of the morphology and blood flow velocity of micro-vessel hotspots in the cervix using transvaginal power Doppler ultrasound (TV-PDU), specifically in patients with LSIL and HPV condyloma, because of the possibility of regression being significantly higher in this category compared to in HSIL group [7]. Moreover, we examined the ability of these parameters to differentiate HPV condyloma and LSIL of the cervix.

## 2. Materials and Methods

### 2.1. Study Design and Patient Population

A total of 50 patients with colposcopic findings of LSIL of the cervix were enrolled at Taipei Veteran General Hospital from January 2016 to December 2018. All colposcopic examinations were performed by one of the authors (P.-H.W.) to avoid interobserver variation after finishing the examination by transvaginal ultrasound. The inclusion criteria were outpatients who underwent colposcopic examination and biopsy due to Pap smear results as LSIL, and the final pathological reports confirmed the diagnosis of LSIL of cervix. Additionally, the inclusive criteria stated that the study subjects should have a positive HPV DNA test. This study excluded non-HPV pelvic infections, or other unrelated LSIL clinical situations, such as cervical myomas, large Nabothian cysts, and other cervical masses. The inconsistent reports of LSILs between Pap smear and final pathological reports were also excluded. Other exclusion criteria included pregnancy, and post-menopausal status. Finally, 26 cases met the inclusion criteria; 13 women with colposcopy-guided-biopsy- and pathologically-confirmed cervical condyloma were included in the current study. A total of 40 women were recruited as the control group, with the inclusion criteria for the control group stating normal cervical cytology, a negative HPV test, normal transvaginal ultrasound of the cervix and uterus, and no abnormal pathology of the cervix, vagina, or uterus upon pelvic examination. The general characteristics of the patients are presented in Table 1. 

### 2.2. Transvaginal Ultrasound with Power Doppler Angiography (TV-PDA)

All 79 women were scanned using a color Doppler ultrasound (Diasonics, Gateway, St Louis, MO, USA) equipped for color Doppler imaging and color Doppler angiography in the semi-lithotomy position with an empty urinary bladder within 24 h of undergoing treatment. A 7.0 MHz curved array endo-vaginal probe was used; the field of view was set to 112 degrees. The uterine cervix was scanned and identified carefully in a longitudinal view in grayscale mode. After shifting to angio mode, the color gain was adjusted from 105 to 115, the scale was adjusted to pulse repetition frequency (PRF) and set from 800–900 Hz to filter out the low-strength signals, and the temporal filter was adjusted to 1.0 to exclude the blue color within the region of interest (box). Vascularity within the uterine cervix was detected in the axial view. We selected the section within the uterine cervix with the most significant blood flow color signals and the most apparent hotspots in the region of interest. The number of hotspots observed under power mode color Doppler ultrasound was classified into three classes (Table 2).

### 2.3. Measurement of Blood Flow Velocity Waveforms within the Uterine Cervix

After visually classifying the grade of vascularity in the power mode color Doppler ultrasound, we activated pulse Doppler mode and adjusted the Doppler gate over the target color area to set the proper gate distance and adjust the angle of the sampling site to less than 60 degrees. We defined the detectable Doppler signals as a series of reproducible, similar arterial waveforms obtained for at least three separate consecutive cardiac cycles. Good Doppler signals were processed and analyzed using online spectra. All measurements were calculated manually, including the peak systolic velocity (PS), end-diastolic velocity (ED), time-averaged maximum velocity (TAMVV), pulsatility index (PI), resistance index (RI), and vascular index (VI). If more than one set of satisfactory samples was achieved, the lowest set of PI, RI, and VI values was recorded. The pulsatility index was defined as the ratio of the difference between the peak systolic and end-diastolic velocity to the mean velocity. The resistance index was defined as the ratio of the difference between the peak systolic velocity and end-diastolic velocity to the peak systolic velocity. The vascular index was defined as the peak systolic velocity divided by end-diastolic velocity (PS/ED). One author (Y.C.W.) performed and assessed all scans to avoid interobserver variation.

### 2.4. Statistical Analysis

The difference in vascular grading between the study groups was analyzed using Fisher’s exact test. Cervical micro-vessel flow waveform parameters were analyzed using the Kruskal–Wallis test. A Post hoc analysis was conducted using the Benjamin–Hochberg procedure. Spearman’s rank correlation was used to assess the relationship between vascular grading and waveform parameters. Clopper-Pearson’s exact method was used to estimate the testing accuracy confidence intervals. The Statistical Package for the Social Sciences (SPSS) for Windows, version 22.0 (IBM Corp., Armonk, NY, USA), was used for all data analysis, including the intra-observer reproducibility and receiver operating curve analysis. *p*-values < 0.05 were considered statistically significant.

## 3. Results

The mean age of the 79 individuals included in this study was 37.8 (23–58 years old). The mean age of the case group (39.9 ± 11.9) and control group (35.8 ± 6.1) was not significantly different (*p* = 0.052). 

### 3.1. Visual Classification of Vascularity Findings on TV-PDU 

**Figure 1:**
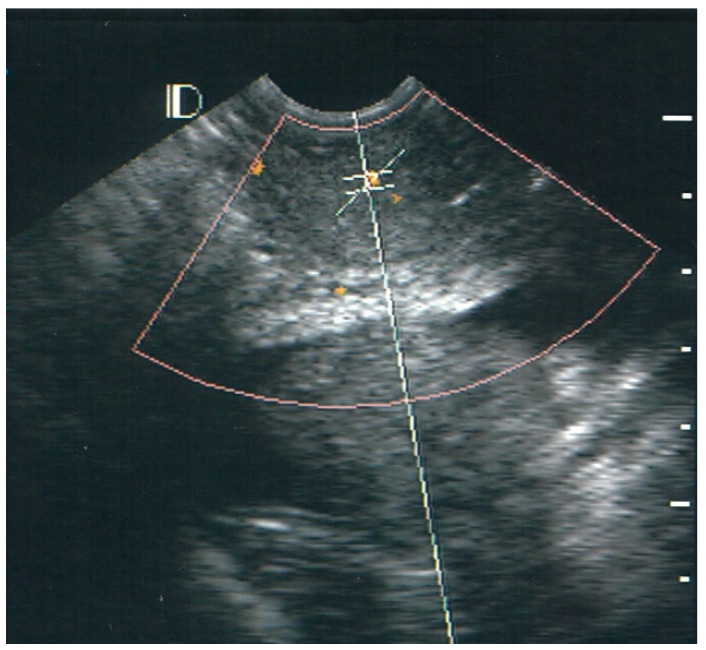
Example of Class I vascularity. There are three vascular hotspots with orange color representing the sagittal section of the uterine cervix area; each size is measured as about 1–2 mm.

**Figure 2:**
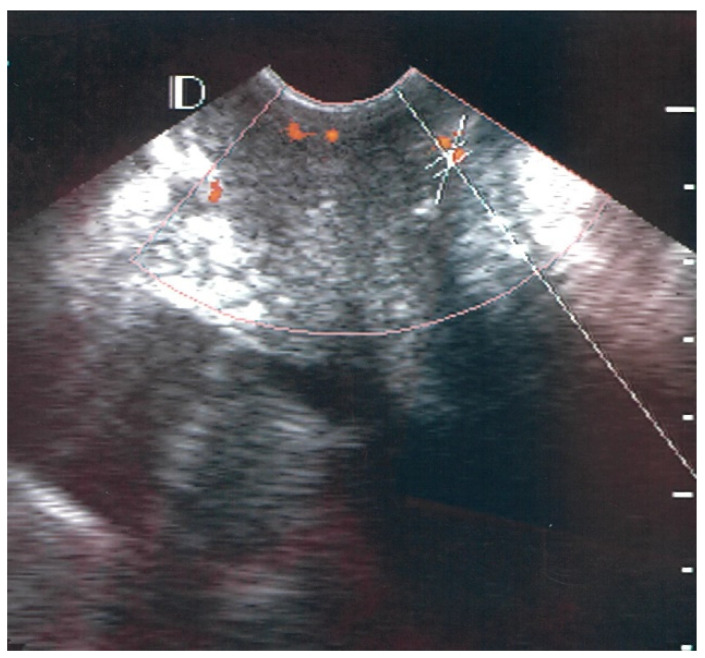
Example of Class II vascularity. There are 4–5 vascular hotspots (orange color) and each size was measured as about 1–2 mm.

**Figure 3:**
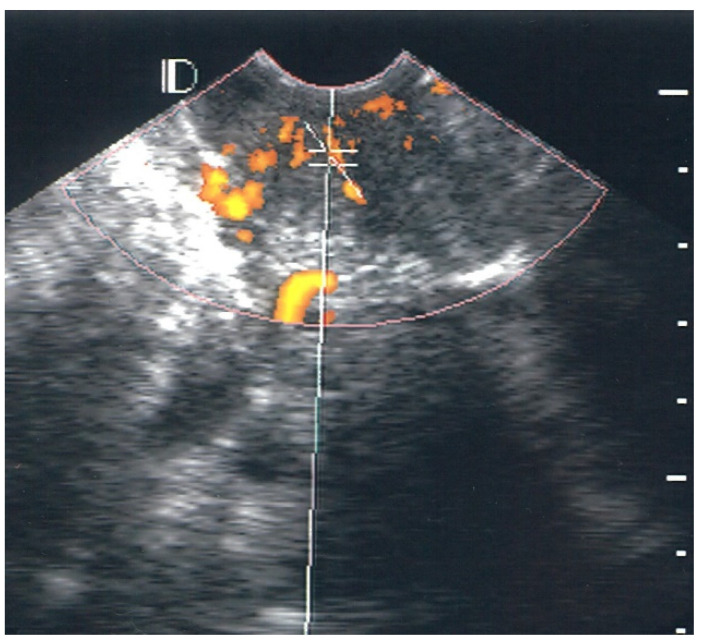
Example of Class III vascularity. There are more than 10 vascular hotspots, and each size ranges from 1 mm to 4 mm.

The pick-up rate for vascularity was 100% (39/39) for the case group and 27.5% (11/40) for the control group. The absence of vascularity was classified as class 0. In the control group, 72.5, 25.0, and 2.5% of cases were classified as class 0, class I, and class II, respectively. No individuals in the control group were classified as class III. Of the 39 patients with LSIL and HPV condyloma of the cervix, 30 (76.9%) cases were classified as class I (Figure 1), 8 (20.5%) as class II (Figure 2), and 1 (2.6%) as class III (Figure 3).

**Figure 4:**
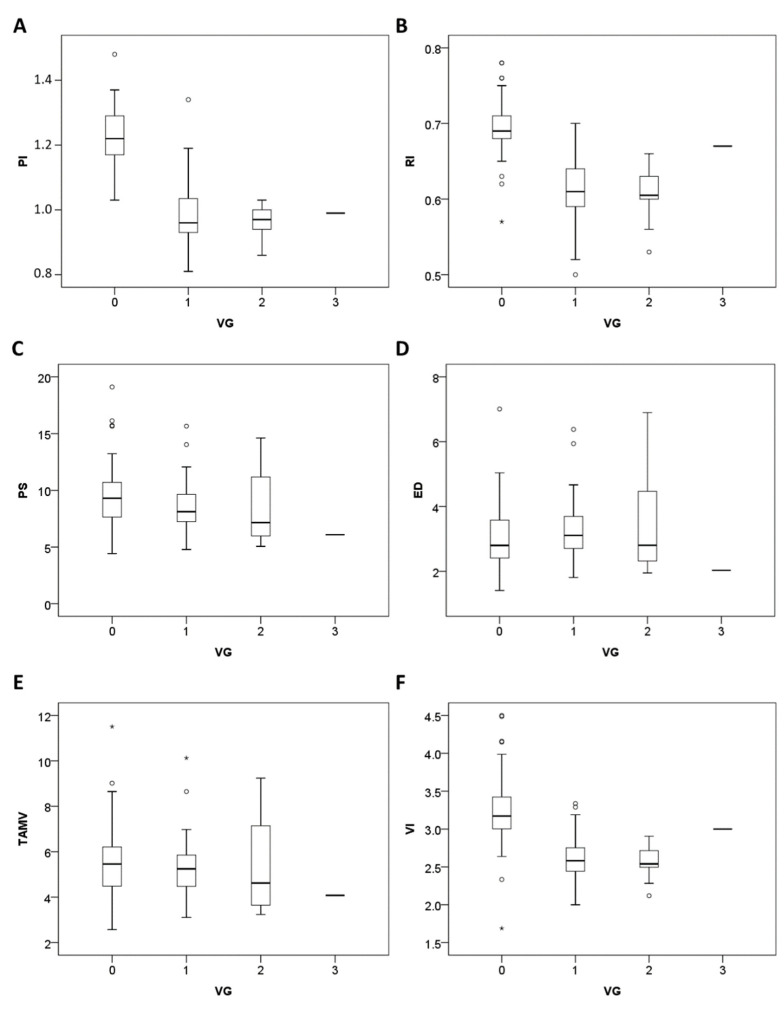
Box plots of the correlations between the vascular grading classification (VG) and six sonographic parameters of the cervix (PI, RI, PS, ED, TAMV, and VI) in the study group (*n* = 39) and control group (*n* = 40). Negative correlations were observed between the VG and the PI, RI, PS, and VI types (**A**,**B**,**C**,**F**); no correlations were observed for ED or TAMV (**D**,**E**). *, Extreme outlier; ∘, Outlier.

According to the visible vascular classification, 12 cases (12/13, 92.3%) of the subgroup with HPV condyloma were classified as class I, 1 (1/13, 7.7%) as class II, and 0 as class III. In the subgroup with LSIL of the cervix, 18 patients (18/26, 69.2%) were classified as class I, 7 (7/26, 26.9%) as class II, and 1 (1/26, 3.9%) as class III. The vascular classification was significantly higher in the subgroup with HPV cervix infection than in the control group (*p* < 0.000). The Fisher exact test with multiple comparison adjustments also revealed significant differences between the HPV condyloma subgroup and control group (*p* < 0.000) and the LSIL subgroup and control group (*p* < 0.000). However, the difference in the vascular classifications of the HPV condyloma subgroup and LSIL (CIN I) subgroup was insignificant (*p* = 0.786). The vascular classification of the cervix correlated negatively with the PI (−0.71, *p* < 0.001), RI (−0.63, *p* < 0.001), VI (S/D)(−0.58, *p* < 0.001), and PS (−0.24, *p* = 0.036), but not with the ED (0.065, *p* = 0.569) or TAMV (−0.105, *p* = 0.357; Spearman’s rho correlation coefficient analysis; Figure 4).

### 3.2. Micro-Vessel Flow Velocity Waveform Findings from TV-PDU

**Figure 5:**
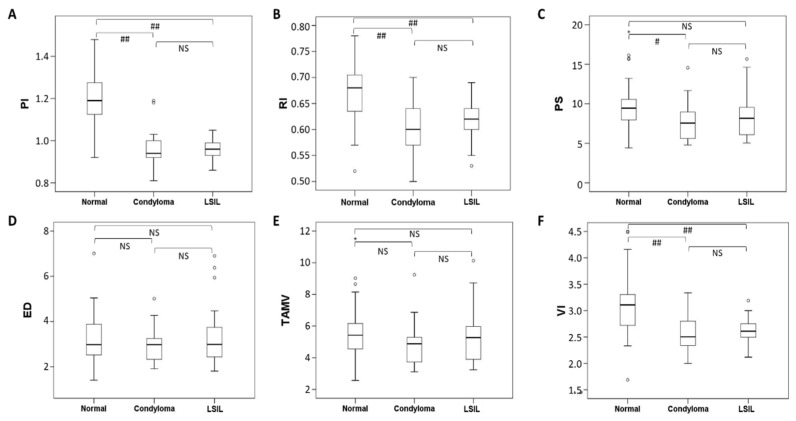
Box plot of the six sonographic parameters (PI, RI, PS, ED, TAMV, and VI) in the HPV condyloma subgroup (*n* = 13), LSIL (CIN I) subgroup (*n* = 26), and control group (*n* = 40). The HPV condyloma and CIN I subgroups had lower PI, RI, and VI than the control group (**A**,**B**,**F**). (**C**) The HPV condyloma subgroup had lower PS than the control group. (**D**,**E**) No significant differences were observed in ED and TAMV between the HPV infection cases (HPV condyloma or CIN I) group and the control group. No significant differences in the six parameters were observed between the HPV condyloma group and LSIL (CIN I) subgroups. * Extreme outlier; ∘, outlier. (##: *p* < 0.01, #: *p* < 0.05, NS: Nonsignificant).

The mean PI was significantly different between the cases (0.96 ± 0.08) and the control group (1.19 ± 0.12; *p* = 0.0000). Similarly, the mean RI was significantly different between the cases (0.61 ± 0.04) and the control group (0.67 ± 0.06; *p* = 0.0000). The mean VI was also significantly different between the cases (2.59 ± 0.30) and controls (3.09 ± 0.58; *p* = 0.0000). Moreover, the PI, RI, and VI differed significantly between the HPV condyloma subgroup and the control group (*p* < 0.01). PI, RI, and VI differed significantly between the LSIL (CIN I) subgroup and the control group (*p* < 0.01; Mann–Whitney U-tests and Kruskal–Wallis tests; Figure 5).

### 3.3. Optimal Cutoff Values for the Six Sonographic Parameters for HPV Condyloma, LSIL (CIN I), and HPV Infection

**Figure 6:**
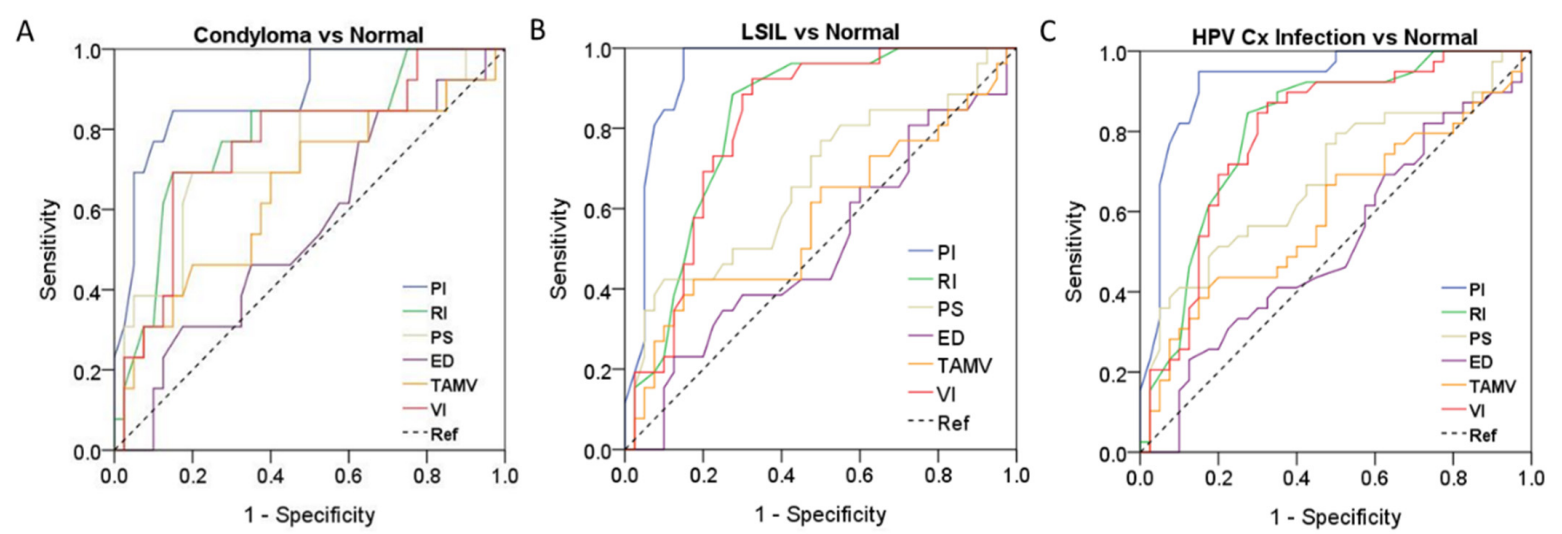
(**A**) ROC curves of the six sonographic parameters for discriminating between HPV condyloma (*n* = 13) and the control group (*n* = 40), (**B**) LSIL (CIN I) (*n* = 26) and the control group (*n* = 40), and (**C**) and HPV infection (*n* = 39) and the control group (*n* = 40).

In the ROC analysis, a PI cutoff value of 1.07 had a sensitivity of 84.6%, a specificity of 85.0%, a positive predictive value (PPV) of 84.9%, a negative predictive value (NPV) of 84.7%, and an accuracy of 88.8% for HPV condyloma. An RI cutoff value of 0.62 had a sensitivity of 69.2%, a specificity of 85.0%, a PPV of 82.2%, an NPV of 73.4%, and an accuracy of 78.7% for HPV condyloma. A VI cutoff value of 2.58 had a sensitivity of 69.2%, a specificity of 85.0%, and an accuracy of 76.3% for HPV condyloma (Figure 6A).

A PI cutoff value of 1.08 had a sensitivity of 100%, a specificity of 85.0%, a PPV of 87%, an NPV of 100%, and an accuracy of 94.2% for LSIL of the cervix. A RI cutoff value of 0.65 had a sensitivity of 88.5%, a specificity of 72.5%, a PPV of 76.3%, an NPV of 86.3%, and an accuracy of 81.9% for LSIL. A VI cutoff value of 2.86 had a sensitivity of 92.3%, a specificity of 67.5%, and an accuracy of 81% for LSIL (Figure 6B).

A PI cutoff value of 1.08 had a sensitivity of 94.9%, a specificity of 85%, a PPV of 86.4%, an NPV of 94.3%, and an accuracy of 92.4% for HPV infection. An RI cutoff value of 0.65 had a sensitivity of 84.6%, a specificity of 72.5%, a PPV of 75.5%, an NPV of 82.5%, and an accuracy of 80.8% for HPV infection. A VI cutoff value of 2.82 had a sensitivity of 84.6%, a specificity of 70%, and an accuracy of 79.4% for HPV infection (Figure 6C).

The PI of the cervix was the most useful sonographic index for distinguishing the micro-vessel flow velocity waveforms of HPV condyloma, LSIL (CIN I), and HPV infections of the cervix (Figure 6 and Table 3).

The intraclass correlation coefficients (ICCs) for all measurements were moderate: the ICC for PI was 0.606 (95% CI, 0–0.893), 0.636 (0–0.902) for RI, 0.638 (0–0.902) for PS, 0.678 (0–0.913) for ED, 0.671 (0–0.911) for TAMV, and 0.649 (0–0.902) for VI.

## 4. Discussion

Bacterial and viral pelvic infections are significant diseases among young and adult females; they affect the female reproductive organs, including the vagina, cervix, womb, fallopian tubes, and ovaries. HPV is the most common viral infection of the reproductive tract and a leading sexually transmitted infection worldwide [16,17]. The vascular resistance (PI, RI) of the uterine arteries, ovarian arteries, and tubo-uterine arteries was low at the beginning of the acute phase of pelvic infection [18,19,20]. These indices rapidly increased and returned to normal after one week and remained constant until one month [18,19,20].

Our analyses based on a visual classification system reveal that most cases of HPV infection presenting as cervical condyloma were classified as class I (12/13, 92.3%), and most cases of LSIL were classified as class I or class II (25/26, 96.2%). The hotspots were not detected in most of the control group members (29/40, 72.5%), and only 10/40 (25%) of the control group were classified as class I. Thus, if the classification is greater than or equal to class I, the case can be regarded as HPV infection of the cervix. The sensitivity of the classification system for detecting HPV infection (LSIL+HPV condyloma) was 100%, and the specificity was 72.5% (Table 1). Thus, the vascular hotspot classification system represents a simple, feasible, non-time-consuming measurement tool for assessing HPV infections in the cervix compared to three-dimensional ultrasound [21,22,23,24,25,26,27,28,29], which requires complete reconstructions.

Belitsos et al. found that three 3D-power Doppler ultrasound indices (VI, FI, and VFI) were significantly higher in a group with precancerous lesions than in a control group (*p* < 0.001) [21]. In their study, the most precancerous lesions were CIN I (34/61, 55.7%), and FI exhibited the most significant differences between the precancerous lesions and the controls. However, the FI is poorly related to flow velocity or the volume of flow [28]. Schulten-Wijman et al. discussed how, even when small vessels are involved, the ability to measure VI and FI at different flow velocities in the clinical setting was highly dependent on the pulse repetition frequency (PRF) and wall motion filter (WMF) settings. Additionally, VI was easily overestimated relative to the actual VI. Thus, they concluded that 3D-power Doppler ultrasound could not be used to measure vascularity in patients with cervical infections [30].

As in our previous study, we assessed the main sonographic parameters in patients with pelvic inflammatory disease presenting as acute cervicitis [31]. Three major Doppler parameters—PI, RI, and VI—were significantly different between the group with HPV cervix infections and the control group. We achieved similar results for different blood vessels: the pick-up rate was 100% (39/39), and the mean PI (0.96 ± 0.08) was significantly lower in the HPV infection group than in the controls. The first main finding of this study is that the vascular classification of cervical hotspots correlates negatively with PI, RI, VI (S/D), and PS. However, no correlations were observed between the classification and ED or TAMV (Figure 4). It is reasonable that infected cervical tissue is susceptible to the induction of angiogenesis. Thus, a lower index value in spectral Doppler analysis would lead to a higher vascular classification. The second finding of this study is that the PI is a more useful parameter than RI, VI, or PS for the differentiation of HPV condyloma and LSIL (CIN I), from healthy controls (*p* < 0.01; Figure 5). 

Dogan et al. [10] reported two prominent findings concerning cervical vascularity. First, there was no significant correlation between the PI of the cervical arteries in a positive high-risk HPV group and a control group (*p* > 0.05). This finding may be because the representative axial Doppler sections did not have significant vascularity, or the control group was not completely healthy but only tested negative for HPV infection. Their second finding was a significantly lower RI for the cervical arteries (0.66 ± 0.86) in the positive high-risk HPV group than in the control group (0.70 ± 0.06) [14]; this result is similar to our study [(0.61 ± 0.04) vs. (0.67 ± 0.06)]. Thus, our visual vascular classification system based on micro-vessel flow velocity waveforms is a simple and rapid method that can also enable the complete quantitative analysis of the cervical tissue in patients with HPV infection [31,32], especially patients with cervical LSIL or HPV condyloma.

There is no doubt that there are some limitations in the current study, including a small sample size, absence of consideration of the potential influence of the menstrual cycle, and no further specific tests to exclude other possibilities of infection. Additionally, we did not perform any additional study to exclude the possibility of concealed underlying diseases, which may influence peripheral circulation. However, by history taking and physical examination, all subjects in the current study appeared “normal” and “healthy”, except those study subjects who had problems with HPV condyloma or LSIL of the cervix. Additionally, in standard routine practice at our hospital, a Pap smear and colposcopic examination are often recommended for premenopausal women when the menstruation period has ended. 

## 5. Conclusions

If the vascular classification is greater than or equal to class I, the sensitivity of the classification system for detecting HPV infection (LSIL+HPV condyloma) was 100%, and the specificity was 72.5%. For cervical HPV infections, a PI cutoff of 1.08 had a sensitivity of 94.9% and a specificity of 85.0% (AUC, 92.0%). Thus, the cervical PI may represent a valuable index for detecting micro-vessel flow waveforms in HPV infections, especially in LSIL and HPV condyloma. This study may provide an alternative and practical method for diagnosing cervical HPV infections using transvaginal ultrasound, although further validation will be needed due to the small sample size in the current study.

## Figures and Tables

**Table 1:** The characteristics of the case group and control group in low-grade squamous intraepithelial (LSIL) and HPV condyloma by TVS-PDU.

Vascular Classification					
	Number	Class 0	Class I	Class II	Class III
LSIL	26	0	18	7	1
HPV condyloma	13	0	12	1	0
Normal	40	29	10	1	0

TV-PDU: Transvaginal Power Doppler Ultrasound, HPV: Human Papilloma Virus.

**Table 2:** Visualize vascular hotspot classification system.

Classification	Numbers of Visualize Vascular Hotspots within Cervix (One Spot: 1 × 1 mm)
Class 0	0
Class I	1 to 5
Class II	5 to 10
Class III	>10

**Table 3:** Optimal cutoff values of the six sonographic parameters for discriminating (A) HPV condyloma and the control group, (B) LSIL (CIN I) of the cervix and the control group, and (C) HPV infection and the control group.

**(A) HPV Condyloma (*n* = 13) vs. Control Group (*n* = 40)**								
Variable	Area	Optimal	Sensitivity	95%LCL	95%UCL	Specificity	95%LCL	95%UCL
PI	0.89	1.07	84.6	54.6	98.1	85	70.2	94.3
RI	0.79	0.62	69.2	38.6	90.9	85	70.2	94.3
PS	0.73	7.68	69.2	38.6	90.9	80	64.4	90.9
ED	0.55	3.52	84.6	54.6	98.1	32.5	18.6	49.1
TAMV	0.65	5.34	76.9	46.2	94.9	52.5	36.1	68.4
VI	0.76	2.57	69.2	38.6	90.9	85	70.2	94.3
**(B) LSIL (CIN I) of the Cervix (*n* = 26) vs. Control Group (*n* = 40)**								
Variable	Area	Optimal	Sensitivity	95%LCL	95%UCL	Specificity	95%LCL	95%UCL
PI	0.94	1.08	100	86.7	100	85	70.2	94.3
RI	0.84	0.65	88.5	69.8	97.6	72.5	58.8	87.3
PS	0.67	7.1	42.3	23.4	63.1	90	76.3	97.2
ED	0.52	2.3	23.1	8.9	43.6	87.5	73.2	95.8
TAMV	0.6	4.48	42.3	23.4	63.1	82.5	67.2	92.7
VI	0.83	2.86	92.3	74.9	99.1	67.5	50.9	81.4
**(C) HPV Infection (*n* = 39) vs. Control Group (*n* = 40)**								
Variables	Area	Optimal	Sensitivity	95%LCL	95%UCL	Specificity	95%LCL	95%UCL
PI	0.92	1.08	94.9	82.7	99.4	85	70.2	94.3
RI	0.81	0.65	84.6	69.5	94.1	72.5	58.8	87.3
PS	0.68	7.68	51.3	34.8	67.6	80	64.4	90.9
ED	0.52	2.33	23.1	11.1	39.3	87.5	73.2	95.8
TAMV	0.6	4.5	43.6	27.8	60.4	80	64.4	90.9
VI	0.79	2.82	84.6	69.5	94.1	70	63.5	83.4

PI: pulsatility index, RI: resistance index, PS: peak systolic velocity, ED: end-diastolic velocity, TAMV: time average maximum velocity, VI: PS/ED, LCL: lower confidence limit, UCL: upper confidence limit.

## Data Availability

The Excel data used to support the findings of this study were supplied by Yi-Cheng Wu under license, and requests for access to these data should be made to Dr. Yi-Cheng Wu (wu102007@gmail.com).
